# GmcA Is a Putative Glucose-Methanol-Choline Oxidoreductase Required for the Induction of Asexual Development in *Aspergillus nidulans*

**DOI:** 10.1371/journal.pone.0040292

**Published:** 2012-07-05

**Authors:** Oier Etxebeste, Erika Herrero-García, Marc S. Cortese, Aitor Garzia, Elixabet Oiartzabal-Arano, Vivian de los Ríos, Unai Ugalde, Eduardo A. Espeso

**Affiliations:** 1 Department of Applied Chemistry, Faculty of Chemistry, University of The Basque Country. Manuel de Lardizabal, San Sebastian, Spain; 2 Department. Medicina Celular y Molecular, Centro de Investigaciones Biológicas (CSIC), Ramiro de Maeztu, Madrid, Spain; 3 IKERBASQUE, Basque Foundation for Science, Plaza Bizkaia, Bilbao, Spain; University of Nebraska, United States of America

## Abstract

*Aspergillus nidulans* asexual differentiation is induced by Upstream Developmental Activators (UDAs) that include the bZIP-type Transcription Factor (TF) FlbB. A 2D-PAGE/MS-MS-coupled screen for proteins differentially expressed in the presence and absence of FlbB identified 18 candidates. Most candidates belong to GO term classes involved in osmotic and/or oxidative stress response. Among these, we focused on GmcA, a putative glucose-methanol-choline oxidoreductase which is upregulated in a Δ*flbB* background. GmcA is not required for growth since no differences were detected in the radial extension upon deletion of *gmcA*. However, its activity is required to induce conidiation under specific culture conditions. A Δ*gmcA* strain conidiates profusely under acid conditions but displays a characteristic *fluffy* aconidial phenotype in alkaline medium. The absence of asexual development in a Δ*gmcA* strain can be suppressed, on one hand, using high concentrations of non-fermentable carbon sources like glycerol, and on the other hand, when the cMyb-type UDA TF *flbD* is overexpressed. Overall, the results obtained in this work support a role for GmcA at early stages of conidiophore initiation.

## Introduction

*Aspergillus nidulans* is a widely used model organism for industrially or medically important filamentous fungi as well as for the study of basic developmental processes in eukaryotes [1]. Since its discovery for science, *Aspergillus* has been exploited for more than six decades to explore fungal genetics and cell biology [2]. It is currently the reference organism in the study of asexual development [3]–[5].

The life cycle of *A. nidulans* starts with the germination of spores, forming vegetative hyphae that extend apically through the deposition of new material at the tip [6]. This vegetative mode of growth is maintained under optimum nutritional and environmental conditions but the exposure of the mycelium to an air interphase [3], [7], light [8], [9] and/or nutrient starvation [10], [11] may activate different signaling pathways which transduce these signals into intracellular cues, ultimately resulting in the activation of *brlA* expression. *brlA* is the master gene for the production of asexual reproductive structures called conidiophores (see references within [4], [5], [12]). Generation of a conidiophore comprises the ordered formation of six well differentiated cell types: the foot-cell, the stalk, the vesicle, primary sterigmata (metulae), secondary sterigmata (phialides) and long chains of asexual spores (conidia) [13].

Some of the genes whose products are involved in the transduction of environmental signals and the activation of the asexual development process have been previously identified ([3], [5] and references therein). Loss-of-function mutations in these genes yield a “*fluffy”* aconidial phenotype that is manifested as masses of vegetative cells and the absence of cell differentiation. From the genetic point of view, the *fluffy* phenotype is associated with the inability to induce the expression of the C2H2-type transcription factor *brlA*, the first conidiation-specific TF [3]. Hence, those regulatory elements acting at this level are generally known as Upstream Developmental Activators (UDA; [5]). FluG is an UDA factor necessary for the synthesis of the terpene dehydroaustinol [14]. This compound, assisted by the orsellinic acid derivative diorcinol, is thought to be required to inhibit the repressive effect on conidiation of the transcription factor (TF) SfgA [15] and consequently activate a set of inducers of development. The bZIP-type TF FlbB and its partner FlbE form a complex at the *Spitzenkörper* of vegetative cells [16], [17], where they could play a sensory function [16], [18]. Jointly with FlbB, the cMyb-type TF FlbD binds the *brlA* promoter and activates asexual development [19]. The C2H2-type TF FlbC activates *brlA* expression through a pathway parallel to that defined by FlbB and FlbD [20].

The understanding of the molecular mechanisms underlying the asexual reproductive process requires a deeper study of the functional relationship among UDA factors as well as the identification of additional regulatory/signalling functions or associated metabolic elements acting at this level. In this study, a 2D-PAGE/MS-MS-coupled screening of proteins with altered cellular levels in the absence of the UDA factor FlbB revealed that one of them was GmcA, a predicted glucose-methanol-choline oxidoreductase. In addition, our results show that GmcA is required in the process of induction of asexual development under specific environmental conditions.

## Results

### Identification of Proteins with Altered Concentration and/or Stability in the Absence of FlbB Activity

**Figure 1:**
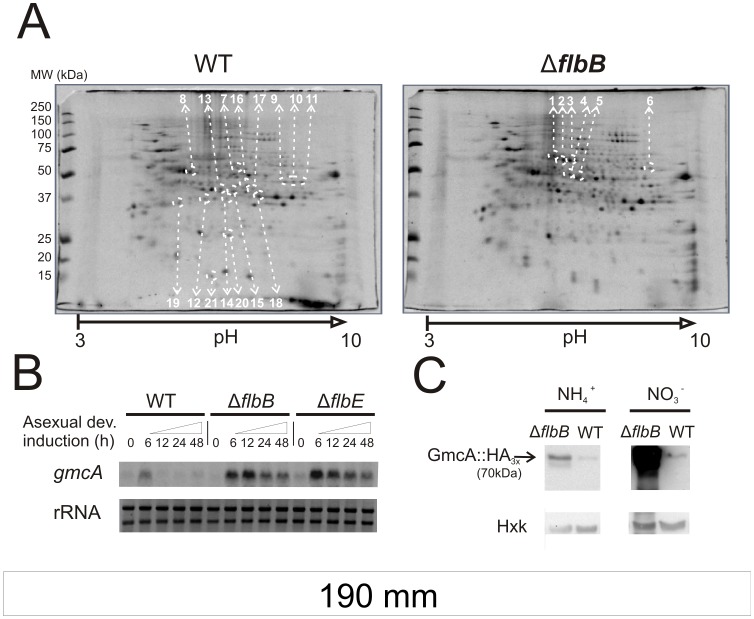
Regulation of *An8547* by the UDA pathway. A) 2D-PAGE images from wild-type (TN02A3) and Δ*flbB* (BD143) strains grown in liquid MMA medium for 18 hours. Every spot analyzed by mass spectroscopy is marked by white dotted circles and the corresponding number. *Locus* identification and GO term classification for proteins in each spot is shown in Table S1. B) Northern blot experiment comparing *An8547* expression levels in the transition of vegetative to asexual development (18 hours of vegetative growth, labeled as 0, and 6, 12, 24 and 48 hours after the induction of conidiation) in the parental wild-type (TN02A3), Δ*flbB* (BD143) and Δ*flbE* (BD142) genetic backgrounds. RNA samples were obtained from mycelia grown using ammonium (10 mM) as the main nitrogen source. Ribosomal RNAs (rRNA) are shown as a loading control. C) Western-blot experiment comparing GmcA-HA_3x_ levels from Δ*flbB* (BD142) and the parental wild type (TN02A3) vegetative mycelia (18 hours), both grown in liquid MMA containing either ammonium (10 mM; left) or nitrate (80 mM; right) as the main nitrogen source. Levels of hexokinase (Hxk) were used as a loading control.

To identify proteins with altered cellular levels in the absence of the UDA factor FlbB, we used the following proteomic approach. Total protein extracts were obtained from mycelia of Δ*flbB* and its parental wild-type, TN02A3, strains and separated using two-dimensional protein electrophoreses (2D-PAGE). Since UDA genes are expressed during vegetative phase and all evidence indicates that they play a role at this stage in the signaling leading to conidiation [4], [5], protein extracts were obtained from vegetative cultures. From more than 200 spots detected in each 2D-PAGE gel, we selected 21 displaying differential intensity: 6 had a higher intensity in the Δ*flb*B strain protein extract (calculated WT/Δ*flbB* volume ratio of those spots, V_WT_/V_Δ*flbB*_, lower than 0.8 in all cases) while 15 had a lower intensity (V_WT_/V_Δ*flbB*_ higher than 1.2). Proteins in those spots were identified by mass spectroscopy with the sole exception of spot-16 (Figure 1A; Table S1). Candidates from searches using the *A. nidulans* protein database yielded high scores, in the range of 238 and 1090. The identified peptides from each spot covered between 41 and 91 percent of their respective candidate sequences.

GO term classification for each candidate is shown in Table S1. In the protein extract from a null *flbB* strain we found reduced amounts of a number of enzymes that participate in glycolytic and gluconeogenic reactions (spots 8, 12, 15 and 20), reduced levels of the NADP+-dependent glycerol dehydrogenase (spot-14), involved in glycerol and arabinose metabolism, and of three enzymes that catalyze different reactions from pentose metabolism (spots 13, 19 and 21). Finally, spots 9, 10 and 11 corresponded to different isoforms of citrate synthase and spots 7 and 18 were related to enzymes involved in amino acid metabolism.

Among candidates directly or indirectly repressed through FlbB regulatory activity, spots 1 and 5 corresponded to enzymes from galactose metabolism and the pentose cycle, respectively, while spots 2 and 3 were identified as a pyruvate decarboxylase. Spot-6 is a NADP-specific glutamate dehydrogenase involved in the synthesis of glutamine-derived amino acids. These results suggest that the presence of FlbB in the cell has an influence in the pattern of carbon metabolism during vegetative growth, from glycolytic to gluconeogenic pathways. This influence extends to amino acid biosynthesis. The above mentioned proteins detected in our assay quantitatively represent major metabolic processes, rather than those elements which are expressed in small amounts or are restricted to specific compartments. For example, we could not detect FlbD among the above proteins, despite the fact that this TF has been proven to be directly regulated by FlbB [19].

Among the proteins discovered in this screen, we detected one with hitherto unreported importance in the induction of conidiation. The product of gene An8547 (corresponding to spot-4) predictably encodes a glucose-methanol-choline oxidoreductase (hereafter referred to as GmcA) with a proposed role in stress response [21], [22].

### UDA Pathway Regulates *AN8547/gmcA* Expression

GMC proteins are, in general, involved in the oxidation of aromatic and aliphatic alcohols to aldehydes. Usually oxygen, O_2_, acts as an electron acceptor and the reaction generates H_2_O_2_ as a product [23]. Interestingly, air exposure is one of the main stimuli that induces development in *Aspergillus nidulans* [3] while reactive oxygen species (ROS) like H_2_O_2_ have widely been linked to development in fungi [24], [25]. In addition, members of the GMC family of enzymes are implicated in the control of diverse aspects of development in higher eukaryotes (see discussion; [26], [27]). Thus, we decided to investigate the role of *An8547/gmcA* in *A. nidulans* conidiophore development. Firstly, we confirmed by Northern-blot experiments that *An8547/gmcA* expression is dependent on FlbB function, and that of its interacting UDA partner FlbE, since both act in a concerted manner [17]. In a wild-type background, *gmcA* transcript levels are low in vegetative hyphae, increase six hours after the induction of conidiation and return to basal levels again as conidiophore development proceeds (Figure 1B). We noticed higher *gmcA* levels at the same developmental stage in the absence of either element of the FlbB/FlbE apical complex that regulates asexual reproduction. Moreover, there was no return to basal expression levels thereafter. Consistent with the accumulation of *An8547/gmcA* transcript in *flbB* or *flbE* null backgrounds, Western blots showed increased levels of a GmcA-HA_3x_ tagged protein in a null *flbB* background (Figure 1C). These results confirm the data from the 2D-PAGE screen and suggest that *gmcA* expression is heightened and mis-scheduled when specific UDA activities are lost. Modulation of *gmcA* transcript levels during early conidiogenesis points to a specific role for this putative oxidoreductase in the asexual differentiation program.

### *An8547* Codes for a Novel Fungal Specific GMC Oxidoreductase

Prior to evolutionary and domain architecture analyses, we confirmed that the *gmcA* cDNA sequence matched that predicted at the Aspergillus Genome Database (http://www.aspgd.org/; NCBI GenBank accession number CBF80774). The cDNA sequence was deposited at the GenBank database with the accession number JN872213.

**Figure 2:**
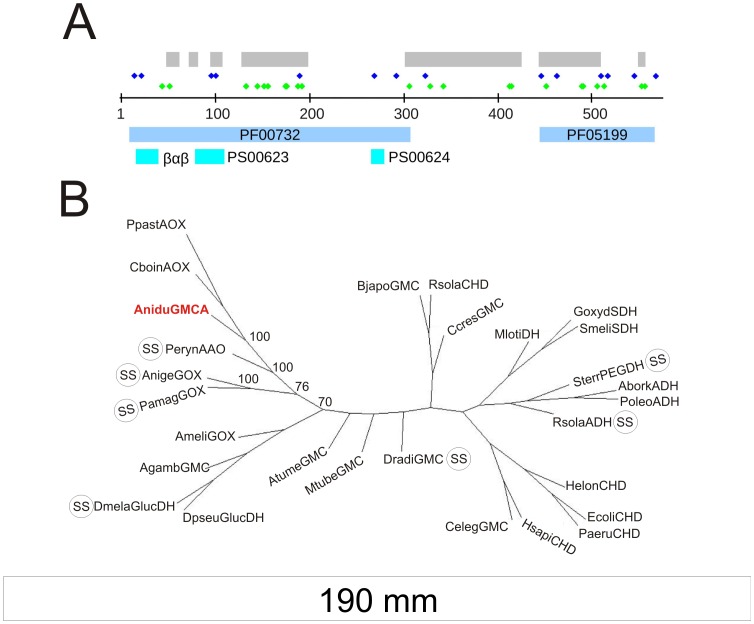
Characterization of *Aspergillus nidulans* GmcA sequence. A) Domain, conserved motif location and residue enrichments in *A. nidulans* GmcA sequence. Gray: Sequence comprising the SBD of GmcA as identified by homology modeling of the GmcA sequence on the PDB 3FIM structure (PerynAAO). Dark blue diamonds: Cysteine residues. Green diamonds: Histidine residues. PF00732 and PF05199: GMC N- & C-term Pfam motifs, respectively. Light blue: Prosite (PS) motifs and the βαβ dinucliotide binding motif. B) Inferred unrooted phylogenetic tree of 27 GMC sequences from the first clade of [23] plus the *A. nidulans* GmcA sequence. GMCs with predicted signal sequence are labeled with SS. Bootstrap support values calculated from 100 trees are indicated at relevant nodes.

GmcA, a 576 amino acids polypeptide, encodes the two Pfam domains [28] that define the GMC family: pfam05199 (GMC oxred C) and pfam00732 (GMC oxred N) at E values of 8.4^−40^ and 7.1^−65^, respectively (Figure 2A). Additionally, the sequence also encodes all defining residues of Prosite motif PS00624 (GMC_OXRED_2), 10 of the 12 defining residues of Prosite PS00623 (GMC_OXRED_1) and the ADP-binding beta-alpha-beta fold involved in FAD binding [29], [30]. BLAST searches revealed high similarities with a large number of GMCs in the NCBI nr database. Restricting the search to Protein Data Bank proteins revealed that GmcA had high homology to several proteins from GMC clade 1 described by [23], which includes, among other types, alcohol and glucose oxidases and alcohol, glucose, sorbose and choline dehydrogenases. The phylogenetic analysis of the 27 clade 1 GMCs plus GmcA resulted in a phylogenetic tree that placed GmcA next to an aryl-alcohol-oxidase (PerynAAO; Table 1; [31]) and between CboinAOX and AnigeGOX (alcohol and glucose oxidases, respectively; Figure 2B; [32]).

**Table 1:** Zamocky Clade 1 GMCs plus *A. nidulans* GmcA.

Abbreviation	NCBI Accession N.	Species	References used in [23] Abbreviation	Accession*
AniduGMCA	CBF80774	*Aspergillus nidulans*	**–**	**–**
AtumeGMC	NP_396582	*Agrobacterium tumefaciens*	Atumef GMC	(NP_536181)
AborkADH	CAC38030	*Alcanivorax borkumensis*	Aborkum ADH	
AmellGOX	BAA86908	*Apis mellifera*	Amellifera GOX	(AB022907)
AnigeGOX	AAA32695	*Aspergillus niger*	Aniger GOX	(J05242)
AgambGMC	EAA08043	*Anopheles gambiae*	Anopheles putative ORF	**–**
BjapoGMC	NP_769660	*Bradyrhizobium japonicum*	BradyjaGMC	**–**
CboinAOX	Q00922	*Candida boinidii*	Cboinidii AOX	**–**
CcresGMC	AAK22929	*Caulobacter crescentus*	CcresGMC	**–**
CelegGMC	Q18429	*Caenorhabditis elegans*	Celegans GMC	**–**
DmelaGlucDH	NP_477503	*Drosophila melanogaster*	Dmelanogaster GlucDH	(NM_058155)
DpseuGlucDH	AAA28572	*Drosophila pseudoobscura*	Dpseudoobscura GlucDH	(M29299)
DradiGMC	NP_294689	*Deinococcus radiourans*	DradiGMC	**–**
EcoliCHD	NP_414845	*Escherichia coli*	Ecoli CHD	**–**
GoxydSDH	BAA13145	*Gluconobacter oxydans*	Glucoxyd SDH	**–**
HelonCHD	CAB77176	*Halomonas elongata*	Helongata CHD	(Q9L4K0)
HsapiCHD	NP_060867	*Homo sapiens*	Hsapiens CHD	(XP_040608)
MlotiDH	NP_102692	*Mesorhizobium loti*	Mloti DH	**–**
MtubeGMC	NP_335763	*Mycobacterium tuberculosis*	MtuberGMC	**–**
PamagGOX	AAD01493	*Penicillium amagasakiense*	Penicillium GOX	(AF012277)
PerynAAO	O94219	*Pleurotus eryngii*	Pleurotus AOX	(AAF31169)
PpastAOX	XP_002493556	*Pichia pastoris*	Ppastoris AOX	(P04842)
PoleoADH	Q00593	*Pseudomonas oleovorans*	Pseudomonas ADH	**–**
PaeruCHD	NP_254059	*Pseudomonas aeruginosa*	Pseudomonas CHD	**–**
RsolaADH	NP_518244	*Ralstonia solanacearum*	Rsolananac ADH	**–**
RsolaCHD	CAD17133	*Ralstonia solanacearum*	Rsolan CHD	**–**
SmeliSDH	AAK65431	*Sinorhizobium melioti*	Smelioti SDH	**–**
SterrPEGDH	BAB61732	*Sphingopyxis terrae*	Sterrae PEG-DH	**–**

* Referenced in [23] but have since been superseded.

**Figure 3:**
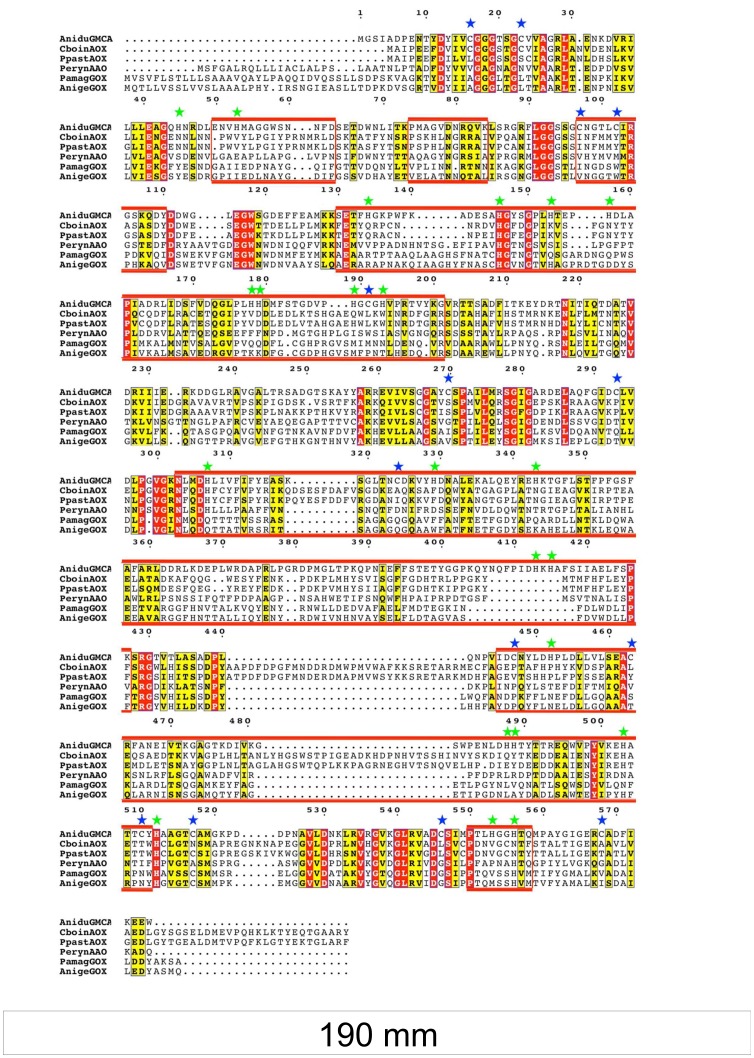
Alignment of GmcA sequence with the five most closely related GMCs based on phylogenetic analysis. Invariant residues are displayed in red background while conserved residues are in yellow. The non-contiguous sequence comprising the substrate binding domain is boxed in red. Table 1 gives the reference for each sequence. Cysteine and histidine residues are marked with blue and green stars, respectively. Residue numbering is for the *Aspergillus nidulans* GmcA sequence.

More detail of the sequence divergence between GmcA and related sequences was sought by aligning the AOX and AAO sequences along with the two most closely related GOX proteins. As the FAD cofactor binding domain is highly conserved across all GMCs while the substrate binding domain (SBD) is highly variable, the *A. nidulans* GmcA sequence was partitioned into the cofactor and substrate binding domains (Figure 2A). After alignment of the six sequences, the sequence blocks comprising the SBD were boxed and residue identity and similarity were highlighted (Figure 3). The alignment clearly shows that substantial differences exist in the SBD regions of the four different types of GMCs. These differences range from residue conservation to insertions and/or deletions. Furthermore, composition analyses comparing GmcA to other known GMCs revealed that GmcA sequence is enriched in cysteines and that the SBD contains 20 of the 22 histidines in GmcA (see Text S1 and Table S2). Six orthologs (among the 26 GMC sequences with BLAST E values of zero) exhibited positional conservation equal to or greater than 85% for both histidine and cysteine residues. Interestingly, these same six fungal species are predicted to contain a functional FlbB/FlbE complex for the signaling of conidiation [33]. In light of these results, we propose that GmcA defines a new subfamily of GMC proteins restricted to ascomycota, close in evolution to AAO, GOX and AOX proteins, and the substrate of which is likely an alcohol.

GmcA is not predicted by SignalP [34] to contain a signal sequence as do seven of the 27 Zamocky clade 1 GMCs [23], including AnigeGOX, PamagGOX and PerynAAO ([35]; Figure 2B). This suggests that GmcA is not likely to be exported, similarly to PpestAOX and CboinAOX. These *in silico* predictions are supported by the observation of GmcA::GFP chimera dispersed throughout the hypha, without any visible accumulation in defined cellular regions or compartments, and not excluded from nuclei (not shown). The same distribution was exhibited in media with different nitrogen sources and during the formation of conidiophores. We predict an intracellular role for GmcA because a HA_3x_-tagged version was not detected in the culture medium.

### GmcA is Required for the Onset of Conidiophore Genesis

**Figure 4:**
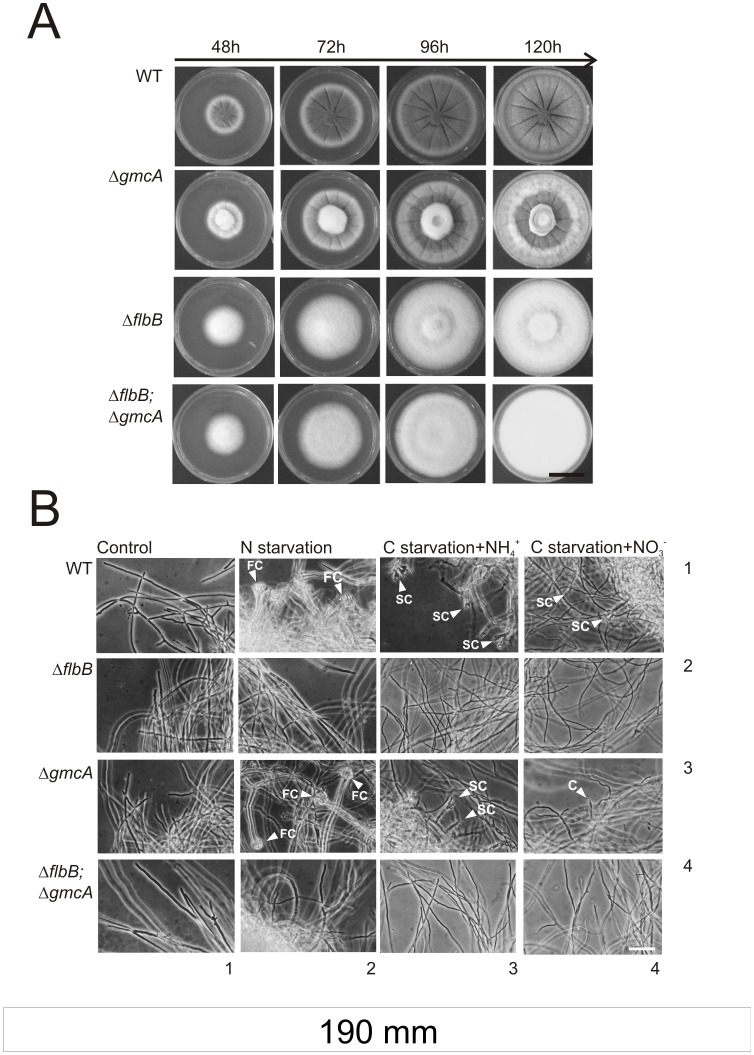
Phenotype characterization of Δ*gmcA* strain on solid and liquid media. A) Colonial growth and conidiation pattern of the Δ*gmcA* (BD429) and Δ*flbB*;Δ*gmcA* (BD431) strains compared to their respective parentals (TN02A3 and BD177, respectively) at 48, 72, 96 and 120 hours of culture on MMA supplemented with nitrate (10 mM) and glucose (1% w/v). Scale bar  = 1.5 cm. B) Nutrient starvation induction of conidiating structures in mycelia from wild-type (row 1), Δ*flbB* (row 2); Δ*gmcA* (row 3) and the double null (Δ*flbB*;Δ*gmcA*) (row 4) strains. Mycelia were cultured for 18 hours at 37°C in MMA and subsequently transferred for additional 20 hours to standard MMA (Control; column 1), MMA without nitrogen (column 2) or MMA without carbon and ammonium (column 3) or nitrate (column 4) as nitrogen sources. FC: Fully developed Conidiophores. SC: Simplified Conidiophores. C: Single conidia emerging from a vegetative cell. Scale bar  = 50 µm.

With the aim of exploring the function of GmcA during the development of asexual structures we generated strains carrying a null allele of *gmcA*, Δ*gmcA*, and the double null Δ*flbB*;Δ*gmcA*. When cultured in standard minimal medium (MMA), containing glucose and nitrate as main carbon and nitrogen sources respectively, we observed that GmcA is not required for radial growth (Figure 4A). However, a Δ*gmcA* mutant showed a visible defect in conidiation compared to an isogenic wild-type strain. This aconidial phenotype is the results of loss of GmcA function, as proved a reconstituted strain carrying an ectopic copy of *gmcA* integrated at the *pyroA locus* (Figure S1). In addition, we verified that this effect is specific for the loss of GmcA activity, since the deletion of *An7832*/*gmcT*, a GMC-family protein with high homology (E  = 2e^−18^, 27% identity, 41% positive over 70% overlap) with respect to GmcA, did not result in a loss of conidiation capability (not shown). This aconidial phenotype of Δ*gmcA* mutants is different in these conditions to that exhibited by a null *flbB* strain and such *fluffy* phenotype is not altered in a Δ*flbB*;Δ*gmcA* double null strain (Figure 4A).

To determine whether GmcA acts at the induction level or during the formation of conidiophore cell types we compared the developmental programs of the parental wild type, Δ*flbB*, Δ*gmcA* and double null (Δ*flbB*; Δ*gmcA*) strains under inducing conditions in submerged cultures. Spores of all strains were initially inoculated and cultured in fully supplemented liquid minimal medium (MMA) and, then, transferred to carbon or nitrogen starvation media, that is lacking either glucose or nitrate (see Experimental procedures; Figure 4B). Consistent with previous studies, nutrient starvation induced the development of complete conidiophores in cultures of the wild type strain, bearing all cell types under nitrogen starvation (FC; Figure 4B) and 2–3 phialides with short chains (SC) of conidia under carbon starvation (SC; Figure 4B; row 1; [11], [36]).

In the Δ*gmcA* strain, nitrogen starvation conditions, using glucose as the main carbon source, induced the production of complete, wild-type like, conidiophores (Figure 4B, column 2; row 3). These results contrasted with the absence of cell differentiation or the production of any kind of conidiating structures by the Δ*flbB* strain (Figure 4B, column 2; row 2; previously described in [36]).

The effect of carbon starvation was studied using two different nitrogen sources: ammonium and nitrate (Figure 4B; columns 3 and 4, respectively). In both growth conditions, the wild-type parental strain produced SCs. However, the Δ*gmcA* strain produced SCs only when ammonium was used while it barely generated single spores arising directly from phialide-like structures [36] when nitrate was used (C; Figure 4B). The double null strain did not produce any type of asexual structures in the conditions studied, suggesting a complete block of asexual developmental program when both *flbB* and *gmcA* were deleted (Figure 4A and 4B).

These results using liquid media show that GmcA is required for the induction of asexual development in nitrate-containing medium, but not for the synthesis of any of the cell types that form the conidiophore. These differences between Δ*gmcA* and Δ*flbB* aconidial phenotypes prompted us to study additional specificities of GmcA activity comparing to the UDA pathway.

### GmcA is Required for the Synthesis of an Extracellular Metabolite Involved in the Induction of Conidiation

**Figure 5:**
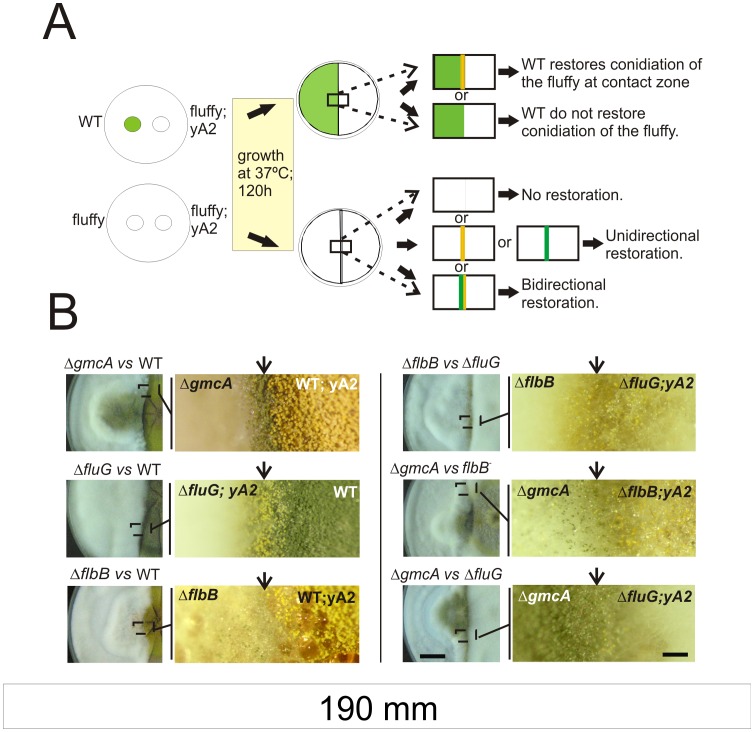
Extracellular complementation assays for conidiation induction. A) Schematic representation of the procedure followed in extracellular complementation experiments and the interpretation of possible results. B) Contact zones and magnifications of selected regions are shown for different combinations of strains. On the left, Δ*gmcA* (BD429; green spores; top), Δ*fluG* (TTA127.4; yellow spores; middle) or Δ*flbB* (BD143; green spores; bottom) null mutants are assayed against the wild type (yellow, MAD782, or green, FGSC26, colored) strains. On the right, contact regions and respective magnifications of selected regions are shown between Δ*fluG* (TTA127.4; yellow) and Δ*flbB* (BD143; green; top), Δ*gmcA* (BD429; green) and *flbB^-^* (BD70; yellow; middle) or Δ*gmcA* (BD429; green) and Δ*fluG* (TTA127.4; yellow; bottom) strains. Images were taken 120 hours after inoculation. Scale bars represent 2 cm (left) and 0.15 cm (right), respectively.

The presence in the medium of certain extracellular metabolites precedes the process of asexual development and these compounds act in trans when produced by different fungal strains [37]. This means that some strains can restore conidiation upon physical contact with specific aconidial mutants (see a scheme of the experiment in Figure 5A and Materials and Methods). To analyze whether GmcA, as described for FluG or FlbB [37], is involved in the synthesis of an extracellular metabolite required to induce conidiation, we checked the restoration of conidiation upon physical contact between Δ*gmcA* and Δ*flbB* strains. We used strains producing different spore colors to easily visualize the trans-induction of asexual development. Complete medium (MCA; see Materials and Methods) supplemented with 80mM nitrate was used in these experiments to achieve an extreme Δ*gmcA* aconidial phenotype (see next section). We found that conidiation of the Δ*gmcA* strain was rescued when in contact with a wild-type strain, similarly to what was determined for Δ*fluG* or Δ*flbB* mutants ([3], [36]; Figure 5B, left block, rows 1, 2 and 3, respectively). We also verified that a Δ*flbB* mutant unidirectionally restored conidiation in the *fluG* loss-of-function mutant, as reported ([36], [37]; Figure 5B; right block, row 1). In addition, no conidiation was observed upon physical contact of two Δ*gmcA* strains (not shown).

Bidirectional restoration of conidiation was observed between a Δ*gmcA* strain in contact with either *flbB^-^* or Δ*fluG* mutants (Figure 5B, right block, rows 2 and 3, respectively). These results suggest that the null *gmcA* strain produces both FluG and FlbB signals, and that the corresponding Δ*fluG* and *flbB-* strains produced the metabolite linked to GmcA activity.

In view of these results, we also tested a Δ*tmpA* strain. The transmembrane protein TmpA participates in the control of conidiation in a different pathway to that defined by FluG [38]. However, we observed again the bidirectional restoration of conidiation when both Δ*tmpA* and Δ*gmcA* mutants were in contact (not shown). Thus, these results suggest that GmcA either does not act in the synthesis of the signaling molecules controlled by the abovementioned genes or it is linked with these synthesis pathways through non-hierarchical relations.

### GmcA Participates in Conidiation at Alkaline Ambient pH

**Figure 6:**
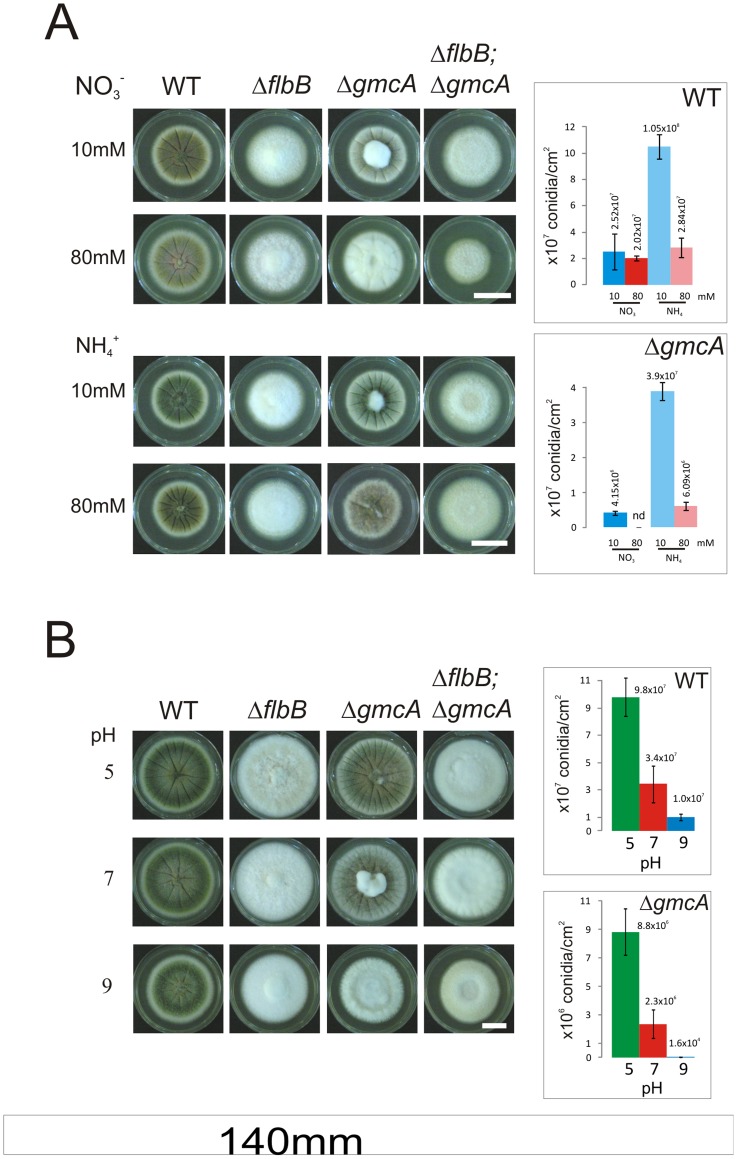
Effect of medium alkalinization on the conidiation capacity of null *gmcA* mutant. A) Conidiation phenotypes after 72 hours of wild-type (TN02A3), Δ*flbB* (BD177), Δ*gmcA* (BD429) and the double null Δ*flbB*;Δ*gmcA* (BD431) strains grown on MMA supplemented with different concentrations of nitrate (upper block) or ammonium (lower block) as main nitrogen sources. Scale bar  = 2 cm. B) Growth of wild-type and mutant strains on MMA with pH values buffered to 5 (upper line), 7 (middle line) and 9 (lower line) at 96 hours of culture in MMA supplemented with urea as the main nitrogen source. Scale bar  = 1.5 cm. Graphs at the right show conidia production per square centimeter for each strain and growth condition.

To determine the environmental context that requires GmcA activity, we examined the conidiation pattern of the Δ*gmcA* strain in solid media of varying compositions (Figure 6A). The conidiation defect of the Δ*gmcA* mutant became more severe at increasing concentration of nitrate (80 mM; Figure 6A; upper panel) but was almost suppressed when ammonium was used as the nitrogen source (Figure 6A; lower panel; see spore production on the right). The Δ*flbB* strain remained *fluffy* with a barely measurable production of conidia in all conditions tested (Figure 6A).

Since nitrate assimilation causes an overall consumption of protons [39] and, hence, alkalinization, we used urea as the main nitrogen source to maintain the growth medium at a constant pH [39]. In these conditions, we analyzed the phenotype of a Δ*gmcA* strain when cultured in media buffered at pH values of 5, 7 or 9. Figure 6B shows that this strain conidiated at a rate 500 fold greater at pH 5 than at pH 9, while the wild type presented a 10 fold difference. We confirmed that the variations in medium pH were minimal (<0.5) after incubation at all the set pH values. These results indicate that GmcA activity is principally required at alkaline pH values (see discussion).

### GmcA Requirement for Conidiation Under Alkaline Conditions is Overcome Using Alternative Carbon Sources to Glucose

**Figure 7:**
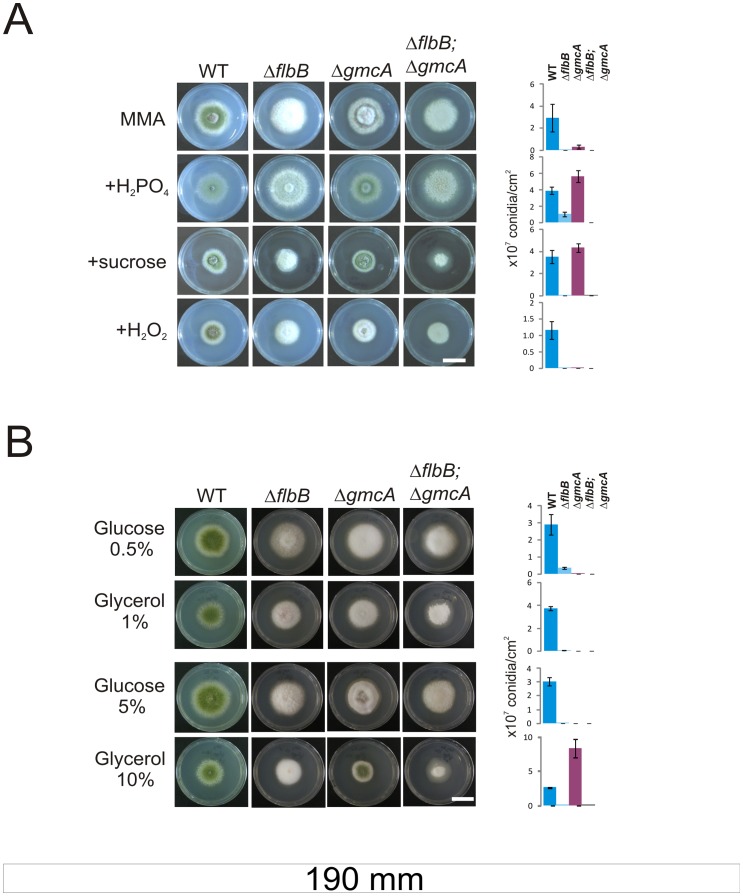
Δ*gmcA* null mutant under different stress conditions or carbon sources. A) Colony and conidiation phenotypes of wild type (TN02A3), Δ*flbB* (BD177), Δ*gmcA* (BD429) and double null (Δ*flbB*;Δ*gmcA*; BD431) cultured on standard nitrate (80 mM) MMA alone or supplemented with H_2_PO_4_ (0.5 M), sucrose (1 M) or hydrogen peroxide (6 mM) after 72 hours of culture. Scale bar  = 2 cm. B) Colonial growth of the same strains in standard nitrate (80 mM) MMA supplemented with different concentrations of glucose (0.5 and 5% w/v) or glycerol (1 and 10%) as carbon sources after 72 hours of culture. Scale bar  = 2 cm. Graphs at the right side show conidia production per square centimeter for each strain and growth condition.

Previous work described transcriptional and translational changes in *gmcA* and the derived protein levels upon menadione exposure [22]. This suggests a putative role of GmcA in the *A. nidulans* response to oxidative stress, and probably additional abiotic stresses. Thus, we followed the Δ*gmcA* phenotype under oxidative but also osmotic and saline stresses. To prevent the use of urea as both carbon and nitrogen sources, we induced medium alkalinization with 80 mM nitrate, conditions in which the null *gmcA* strain exhibits a marked aconidial phenotype. The addition of dihydrogen phosphate as salt-stress inducer (Figure 7A; second column) or sucrose as osmotic-stress inducer (Figure 7A; third column) increased conidia production while the addition of compounds causing oxidative stress such as hydrogen peroxide (Figure 7A; fourth column) or menadione (not shown) did not and neither caused any defect on the growth of the null *gmcA* mutant, so the activity of this enzyme is not essential to mediate response to these stresses for colony growth.

It has recently been shown that the ratio of asexual and sexual structures is altered in a *veA* wild-type background when glucose concentration is increased from 1 to 2% [10]. By comparing the Δ*gmcA* aconidial phenotype in both *veA1* and wild-type *veA* backgrounds with the corresponding isogenic wild-type strains (under constant light or darkness), we observed no phenotypic changes, and concluded that the role of GmcA in the induction of conidiation is not VeA-dependent (not shown). However, we wanted to study the effect of increasing concentrations of different compounds by using glucose as fermentable and glycerol as non-fermentable carbon sources. For a more detailed analysis, carbon sources were added in order to render equimolar carbon concentrations. Nitrate was used again as the nitrogen source instead of urea and the pH of the medium was confirmed to be 8.0±0.5 at the incubation times shown in Figure 7B. In a range glucose concentrations (0.3; 0.5; 1; 2 and 5%) the Δ*gmcA fluffy* phenotype was not altered (lower 0.5% and upper 5% limits of the range shown in Figure 7B, rows 1 and 3, respectively). Δ*gmcA* also remained *fluffy* at 1, 2 and 5% glycerol but produced three times more conidia than the wild type at 10% glycerol (Figure 7B, rows 2 for 1% glycerol and 4 for 10%, respectively). The effect of these specific carbon sources is demonstrated because colonies of a Δ*flbB* strain remained aconidial in all cases. The effect of high glycerol concentrations on the Δ*gmcA* phenotype could be derived from a re-organization of metabolic pathways, eliminating the requirement of GmcA activity to induce *brlA* expression. Alternatively, the high osmolarity induced by the excess of glycerol could provoke on the Δ*gmcA* strain a similar response to that induced by 1M sucrose (Figure 7A).

### *brlA* is Induced in the Δ*gmcA* Strain in Medium with High Glycerol Concentrations

**Figure 8:**
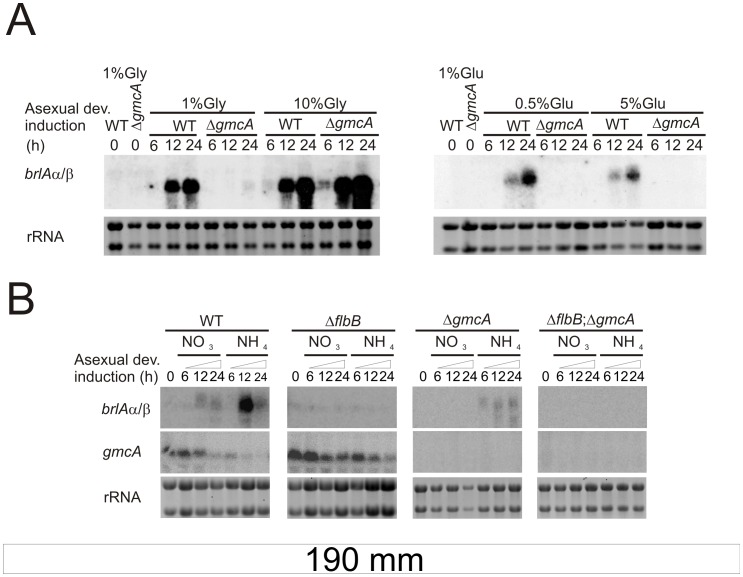
Nitrogen and carbon regulation of *brlA* expression in the Δ*gmcA* mutant. A) Northern blot experiments showing *brlA* expression in wild type (TN02A3) and Δ*gmcA* (BD429) genetic backgrounds during vegetative growth (18 hours), labeled as 0, and 6, 12 and 24 hours after the induction of asexual development. RNA samples were obtained as described in Materials and Methods. Nitrate (80 mM) was used as the main nitrogen source while glucose (Glu) or glycerol (Gly) was used as carbon source. B) *gmcA* and *brlA* expression levels in wild type (TN02A3), Δ*flbB* (BD177), Δ*gmcA* (BD429) and double null (Δ*flbB*, Δ*gmcA*; BD431) backgrounds, at the same time points as in panel A. RNA samples were obtained from mycelia grown in nitrate (80 mM) or ammonium (10 mM)-containing media, using glucose (1%) as the main carbon source. rRNA: Ribosomal RNAs, used as loading control.

The suppression of the Δ*gmcA* aconidial phenotype under specific environmental conditions, i.e. 10% glycerol when 80 mM nitrate is used or 10 mM ammonium when 1% glucose is used, may be interpreted as resulting from the induction of *brlA* expression. To verify this, we analyzed *brlA* mRNA levels under conditions previously shown to promote or repress conidiation in the Δ*gmcA* strain. Firstly, we checked the results shown in Figure 7B. Total RNA samples were extracted from mycelia of wild-type and Δ*gmcA* strains, grown in liquid (vegetative, 18 h, 0 time at figures) or solid (asexual; 6 h, 12 h and 24 h; see Materials and Methods) MMA supplemented with 80 mM nitrate and varying concentrations of glucose or glycerol. Figure 8A confirmed *brlAα/β* expressions in the Δ*gmcA* strain when 10% glycerol was used but *brlA* transcripts were not detected in 0.5 or 5% glucose and 1% glycerol. These results explained the phenotypes shown in Figure 7B.

Secondly, we analyzed the expression levels of *brlAα/β* and *gmcA* at the same time points but, in this case, total RNA samples were extracted from mycelia of wild-type, Δ*flbB*, Δ*gmcA* and double null strains grown in media containing either nitrate (80 mM) or ammonium (10 mM) as the main nitrogen source and glucose (1%) as the carbon source (Figure 8B). As expected, the culture medium was alkaline when nitrate was added and acid when ammonium was used. In samples from the wild type strain, *brlA* expression was lower when nitrate was used (Figure 8B). In samples from the Δ*gmcA* strain *brlA* transcription was reduced in ammonium medium but undetectable in nitrate medium (Figure 8A, third column). This indicates that conidiation is favored in ammonium medium, and that GmcA activity is principally required in nitrate, which correlates with the conidiation levels measured in Figure 6A. The absence of *brlA* transcript in the double null Δ*flbB*;Δ*gmcA* mutant in any of the conditions and times analyzed in Figure 8B accounts for the extremely aconidial phenotype observed in this strain.

### Overexpression of FlbD Suppreses the Lack of GmcA Activity

**Figure 9:**
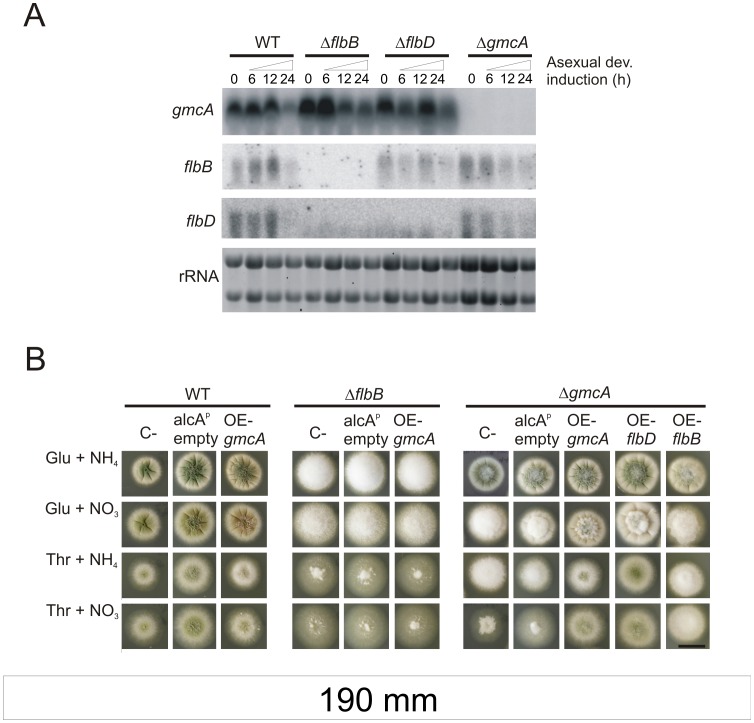
Transcriptional relationship of *gmcA* with the UDA pathway. A) Northern blot experiments showing *flbB*, *flbD* and *gmcA* expression in wild type (TN02A3), Δ*flbB* (BD177), Δ*flbD* (BD198) and Δ*gmcA* (BD429) genetic backgrounds, during vegetative growth and asexual development. Numbers indicate the time (hours) of incubation. RNA samples were obtained from mycelia grown in liquid MMA using nitrate (80 mM) as the main nitrogen source. rRNA: Ribosomal RNAs, used as loading control. B) Phenotypes of strains overexpressing (OE) *gmcA*, *flbB* or *flbD* under the control of *alcA* promoter in wild type (BD520), Δ*flbB* (BD523) or Δ*gmcA* backgrounds (BD526; BD545 and BD543, respectively), in MMA containing 5 g.l^−1^ yeast extract and glucose (Glu, 1%) or threonine (Thr, 100 mM) as the carbon source and ammonium (NH_4_^+^, 10 mM) or nitrate (NO_3_^−^, 80 mM) as the nitrogen source. C_-_ designates parental strains (TN02A3; BD143 and BD429, respectively) and *alcA^p^* empty strains transformed with the empty pALC-pyroA* plasmid (BD188; BD194 and BD531, respectively; [17]). Images were taken 48 hours after inoculation. Scale bar  = 2 cm.

The fact that *gmcA* transcript levels are higher when medium contains nitrate (Figure 8B, first and second columns) supports a hypothesis where GmcA oxidoreductase activity is a pre-requisite for subsequent *brlA* expression at those specific conditions. Thus, we continued the study of the transcriptional relationship of *gmcA* with the UDA pathway using RNA samples obtained from mycelia grown in nitrate (80 mM) medium. Expressions of *gmcA*, *flbB* and *flbD* were then analyzed in wild type, Δ*flbB*, Δ*flbD* and Δ*gmcA* genetic backgrounds (Figure 9A). *gmcA* transcript levels were not altered in a Δ*flbD* background, suggesting that FlbD does not control its expression (Figure 9A, first row). Furthermore, we observed no major alteration in *flbB* and *flbD* expression pattern in a Δ*gmcA* background (Figure 9A, rows 2 and 3, respectively), suggesting that the transcription of both genes is not affected when GmcA is absent.

To gain more information on the transcriptional relationship among these three genes, *alcA^p^* driven over-expression of *gmcA*, *flbB* or *flbD* was studied in different genetic backgrounds (see Materials and Methods; [19]). Threonine (100 mM) was used as inducer of *alcA* expression and over-expression was confirmed by Northern-blot (not shown). Repression of *alcA^p^* was achieved using yeast extract (5 g.l^−1^) and glucose (1%). Phenotypes of recombinant and recipient strains were analyzed under *alcA*-induced and repressing conditions in either ammonium (10 mM) or nitrate (80 mM) solid minimal medium (Figure 9B). Over-expression of *gmcA* did not promote any visible phenotypic alteration in the parental wild type strain (Figure 9B, left block of panels) while it complemented the Δ*gmcA* aconidial phenotype (Figure 9B, right block). In agreement with Northern-blot experiments shown in figure 1B, *gmcA* overexpression did not reverse the Δ*flbB fluffy* phenotype (Figure 9B, middle block), indicating that the partial suppression of the *fluffy* phenotype observed in a null *flbB* strain when cultured for an extended period of time [12], [37] was not due to accumulation of *gmcA* transcript and thus GmcA activity.

Relevant to the understanding of the regulatory mechanisms under the UDA pathway was the finding that conidiation was restored in a null *gmcA* background when *flbD* but not *flbB* was over-expressed. This suggests that GmcA could act upstream to FlbD regulatory activity. However, the genetic interactions between *gmcA* and *flbB* do not allow us to locate the former sequentially downstream of *flbB* in the UDA pathway.

## Discussion

A first study of those proteins presenting altered levels in a Δ*flbB* mutant allowed us to identify GmcA, the first member of a new glucose-methanol-choline oxidoreductase family restricted to ascomycota. There is no evidence supporting *gmcA* as a direct transcriptional target of FlbB. Moreover, neither of the known consensus binding motifs for FlbB were found in the 2000 base-pairs upstream of the GmcA coding region [16], [19]. However, an important issue is that *gmcA* expression is elevated in the absence of FlbB, and yet GmcA is required for conidiophore development. This indicates that the aconidial phenotype of the Δ*flbB* strain is likely due to factors other than the overproduction of GmcA. Miss-scheduled GmcA expression and localization may be the critical factor behind the pattern of observed results.

FlbB exerts a relevant role at early stages of conidiophore development, where it activates *brlA* expression jointly with FlbD [19]. Our results suggest that GmcA may exert its role at this stage of development, since *flbD* overexpression overcomes the aconidial phenotype of Δ*gmcA* mutants. However, GmcA displays some specificity with respect to FlbB activity since, while the Δ*flbB* mutant is aconidial under a wide array of environmental conditions, the Δ*gmcA* strain is *fluffy* only under alkaline conditions.

Alkaline pH constitutes a major environmental stress for *Aspergillus nidulans* and, up to date, we have described three transcription factors whose activities are required for tolerance to alkalinity: PacC mediates the ambient pH regulatory pathway, CrzA mediates the homeostasis of calcium and SltA mediates cation/salt stress response [40], [41]. Interestingly mutations in any of these principal regulatory elements cause major morphological defects including compact colony morphology and reduced conidiation. In fact, CrzA has been directly related with conidiation through the regulation of *brlA* expression [42]. The oxidoreductase GmcA is not required for tolerance to alkalinity neither for cation, salt, ROS nor osmotic stresses and, in agreement with our observations, targets for PacC, SltA or CrzA were not found at the *gmcA* promoter. An indirect mode of control by these factors cannot be ruled out at this stage as the activity of GmcA is required to provide with appropriate asexual development under alkaline growth conditions. The aconidial phenotype of the Δ*gmcA* strain in those conditions resembles that of *fluffy* mutants and we have confirmed that the expression of *brlA* is greatly decreased in this strain. The complete inhibition of *brlA* expression in the Δ*flbB*;Δ*gmcA* mutant and its aconidial phentotype at all growth conditions assayed suggest that GmcA also participates in the induction of development in other growth conditions.

The role of Gmc proteins in the development of higher eukaryotes has been documented but this is the first report in fungi. GMC enzymatic activity is required in important developmental pathways of *Drosophila melanogaster*, such as syntheses of rhodopsins and ecdysteroids [43]–[45]. The Drosophila *ninaG* gene encodes a GMC-type oxidoreductase that catalyzes a late step in the synthesis of the Rh1 rhodopsin chromophore [46], [47]. In insects, one pathway of the metabolism of the moulting and sex hormones ecdysteroids [26], [43], [48], [49] involves the GMC family protein ecdysone oxidase, which catalyzes the oxidation of ecdysone into 3-dehydroecdysteroid [49]. In this work, we demonstrate that GmcA activity in conidiation is specific because it is not shared by GmcT, the closest GMC oxidoreductase within 24 encoded by *Aspergillus* genome. However, the details of the enzymatic reaction catalyzed by GmcA remain to be specified in a forthcoming study.

Asexual development under solid culture conditions involves the contact with the atmosphere, and extracellular metabolites are known to participate in the induction o the process. The UDA FluG is known to control the synthesis of an extracellular triggering signal. Two additional gene clusters also participate in the synthesis and extracellular accumulation in the culture medium of this signal, the meroterpenoid deshydroaustinol [14], [50]. Colony contact experiments clearly demonstrate that GmcA directly or indirectly participates in the biosynthesis of a metabolite which can be transmitted extracellularly. The results indicate that this compound is unrelated to the FluG or FlbB extracellular signaling compounds. The nature of the metabolite is currently under study.

Although we could not identify the reaction catalyzed by GmcA, the requirement of this protein for the induction of development in *Aspergillus nidulans* is evident. It will be the challenge for future studies to unravel the genetic relationship of *gmcA* with the UDA pathway and how the cellular processes that demand the activity of those proteins are coupled to generate a coordinated asexual response, providing the fungus with an enhanced capability for adapting to changing environment.

## Materials and Methods

### Strains, Oligonucleotides and Culture Media

Strains and oligonucleotides used in this study are listed in Table 2 and Table S3, respectively. Liquid and solid standard minimal (MMA) and complete media (MCA: MMA +5g.l^−1^ yeast extract) were used with the appropriate supplements. Nutrient depletion experiments were performed essentially as described previously [36]. Briefly, 10^6^ spores/ml of each strain under analysis were inoculated in liquid MMA and cultured for 18 hours at 37°C and 250 rpm. Mycelia were then filtered and re-inoculated in fully supplemented MMA, MMA without any carbon source or MMA without a nitrogen source. Images were taken after 20 hours of culture in starved media.

**Table 2:** *Aspergillus nidulans* strains used in this study.

Strain	Genotype	Source
TNO2A3	*pyrG89; ΔnkuA::argB; argB2; pyroA4*	[64]
FGSC26	*biA1*	[65]
TTA127.4	*pabaA1, yA2*; Δ*fluG::trpC*	
TGS6	*pabaA1, yA2; ΔargB::trpCDB; trpC801; ΔtmpA::argB; veA1*	[38]
BD70	*pabaA1 yA2; flbB(G456A); ΔargB::trpCDB; trpC801*	[36]
BD142	*pyrG89; ΔnkuA::argB; argB2; ΔflbE::pyrG, pyroA4*	[17]
BD143	*pyrG89; ΔnkuA::argB; argB2; ΔflbB::pyrG, pyroA4*	[36]
BD177	*pyrG89, pabaA1; ΔnkuA::argB; ΔflbB::riboB, pyroA4; riboB2*	[17]
BD188	*pyrG89; ΔnkuA::argB; argB2; pyroA4, alcA(p)::pyroA* *	[19]
BD194	*pyrG89;ΔnkuA::argB;argB2;ΔflbB::pyrG,pyroA4, alcA(p)::pyroA* *	This study
BD198	*pyrG89, pabaA1; ΔnkuA::argB; ΔflbD::riboB, pyroA4; riboB2*	[19]
BD429	*pyrG89;ΔnkuA::argB;argB2;pyroA4;ΔgmcA::pyrG*	This study
BD431	*pyrG89, pabaA1; ΔnkuA::argB; ΔflbB::riboB,pyroA4; ΔgmcA::pyrG; riboB2*	This study
BD438	*pyrG89; ΔnkuA::argB; argB2; gmcA::gfp::pyrG, pyroA4*	This study
BD440	*pyrG89; ΔnkuA::argB; argB2; gmcA::3ha::pyrG, pyroA4*	This study
BD442	*pyrG89, pabaA1; ΔnkuA::argB; ΔflbB::riboB,pyroA4; gmcA::gfp::pyrG; riboB2*	This study
BD444	*pyrG89, pabaA1; ΔnkuA::argB; ΔflbB::riboB,pyroA4; gmcA::3ha::pyrG; riboB2*	This study
BD516	*pyrG89; ΔnkuA::argB; argB2; pyroA4; ΔgmcT::pyrG*	This study
BD520	*pyrG89; ΔnkuA::argB; argB2; pyroA4, alcA(p)::gmcA::gfp::pyroA* *	This study
BD523	*pyrG89; ΔnkuA::argB; argB2; ΔflbB::pyrG,pyroA4, alcA(p)::gmcA::gfp::pyroA* *	This study
BD526	*pyrG89; ΔnkuA::argB; argB2; pyroA4, alcA(p)::gmcA::gfp::pyroA* * *; ΔgmcA::pyrG*	This study
BD531	*pyrG89; ΔnkuA::argB; argB2; pyroA4, alcA(p)::pyroA* * *; ΔgmcA::pyrG*	This study
BD543	*pyrG89; ΔnkuA::argB; argB2; pyroA4, alcA(p)::flbD::pyroA* * *; ΔgmcA::pyrG*	This study
BD545	*pyrG89; ΔnkuA::argB; argB2; pyroA4, alcA(p)::flbB::pyroA* * *; ΔgmcA::pyrG*	This study
BD604	*pyrG89; ΔnkuA::argB; argB2; pBS::pyroA* * *; ΔgmcA::pyrG*	This study
BD605	*pyrG89; ΔnkuA::argB; argB2; pBS*::*gmcA::pyroA* * *; ΔgmcA::pyrG*	This study

* All strains are *veA1*.

Ammonium or nitrate was added at 10 or 80 mM. The standard concentration of all carbon sources was 1% (w/v). Saline, osmotic or oxidative stress conditions were induced by adding sodium dihydrogen phosphate (0.5 M), sucrose (1 M), hydrogen peroxide (6 mM) or menadione (10–60 µM) to nitrate or ammonium-containing MMA. Induction of asexual and sexual development and localization analyses were carried out as previously described [16], [17].

For the generation of the *gmcA::gfp* overexpression plasmid, a genomic DNA fragment containing the *gmcA::gfp* fusion was amplified by PCR from strain BD438 using oligonucleotides flbF-gfpFP-alcA and alcA-gmcA-Up. These oligonucleotides contained EcoRI and BamHI restriction sites, respectively, and allowed the ligation with an EcoRI-BamHI digested pALC-pyroA* plasmid [17]. Plasmids for *flbB* or *flbD* overexpression were obtained previously by Garzia and coworkers [19]. Overexpression experiments driven by the *alcA* promoter were performed in MMA containing 5 g.l^−1^ yeast extract and 100 mM threonine as carbon source (MMT) essentially as described in [17]. Samples for RNA extraction were obtained after the inoculation of 10^6^ spores.ml^−1^ and their culture for 18 hours at 37°C and 250rpm in MMA and subsequent transfer to MMT for 6 hours.

RNA samples for the analysis of *brlA* expression in media with different concentrations of either glucose or glycerol were obtained as follows: 10^6^ spores.ml^−1^ of strains TN02A3 or BD429 were cultured for 18 hours in liquid MMA with 80 mM nitrate and either 1% glucose or 1% glycerol. Mycelia were filtered to induce asexual development and deposited onto solid MMA plates containing 80 mM nitrate and 0.5% or 5% glucose, and 1% or 10% glycerol, respectively. Samples were processed for RNA extraction after 6, 12 and 24 hours of culture at 37°C.

TN02A3, BD177, BD143 and BD429 were used as recipient strains for deletion, tagging and overexpression of the genes of interest. All transformation cassettes for deletion and tagging were generated using the fusion PCR technique [51] and transformation of protoplasts was essentially performed as described by Tilburn and colleagues [52]. Homologous monocopy recombination was confirmed by Southern-blot while overexpression of *gmcA*, *flbB* and *flbD* were verified through Northern blotting.

Strain (BD605) was generated as follows: We amplified a PCR cassette including the *gmcA* ORF plus 1.5 kb from both the upstream and 3′UTR regions (oligonucleotides gmcA-PP1 and gmcA-GSP4). The cassette was inserted between the two NotI sites of pGEM-T-easy vector (Promega). This fragment was released from the pGEM-T-easy vector after digestion with NotI and inserted into a previously generated pBS (pBlueScript SK+, Stratagene) plasmid bearing a truncated pyroA* allele (unpublished), also digested with NotI. We transformed strain BD429 with this recombinant and the empty plasmids and selected single-copy plasmid integrations at the *pyroA locus* through Southern blotting using specific radiolabelled probes.

For extracellular complementation experiments MCA supplemented with nitrate (80 mM) was used. Strains were point-inoculated onto the solid medium at a separation of 2 cm. After 5 days of cultivation, the contact zone was examined and photographed under a binocular Nikon SMZ800 microscope.

To determine spore production in each condition and strain, colony diameter was measured after the incubation time. Spores were collected in 1 ml of an aqueous solution containing 0.02% tween 20 (Acros Organics) and counted. Spore concentration (spores per square centimeter) was determined by dividing spore number with colony area.

### Fluorescence Microscopy

Observation of fluorescent chimeras was carried out using a DMI6000B Leica microscope, equipped with a 63x Plan Apo 1.4 N.A oil immersion lens (Leica), illuminated with a 100w mercury lamp and fitted with a GFP (excitation 470 nm; emission 525 nm) filter. Images were recorded with an ORCA-ER digital camera (Hamamatsu Photonics) and processed with Metamorph (Universal Image) or ImageJ 1.37 (http://rsb.info.nih.gov/ij/) software.

### Preparation of Protein Extracts and Western-blot Analyses

Two different protocols were used for total protein extraction, our standard procedure described in [17], and a modification for *Aspergillus nidulans* of the alkaline lysis extraction procedure used for *Saccharomyces cerevisiae* described in [53]. For the latter, mycelia were collected, frozen in dry ice and lyophilized. Cells were disrupted using a Minibeadbeater (Biospec Products) and 3–5 mg samples were transferred to Eppendorf tubes for solubilization in 1ml/tube of lysis solution (0.2 M NaOH and 0.2% (vol/vol) β-mercaptoethanol). Tubes were vortexed, incubated on ice for 10 minutes, precipitated with 7.5% (vol/vol) trichloroacetic acid and centrifuged at 14,000 g for 5 minutes at 4°C. Pellets were resuspended in 0.1 ml Tris base (1 M), mixed with 2 volumes of Laemmli loading buffer, and incubated for 2 min at 100°C.

Proteins were then resolved in 10% SDS-polyacrylamide gels, electrotransferred onto nitrocellulose filters and exposed to rat anti-HA (Roche; 1/1.000) or rabbit anti-hexokinase (1/80,000) monoclonal antibody cocktails. Peroxidase conjugated anti-rat (Southern Biotech; 1/4,000) or anti-rabbit (Sigma; 1/10,000) IgG immunoglobin were used as secondary antibodies. Peroxidase activity was detected with SuperSignal® West Pico Chemiluminiscent Substrate (Thermo Scientific).

### Precipitation of Protein Extracts, Two-dimensional Electrophoresis and Image Acquisition

Samples containing 200 µg of protein were precipitated using methanol/chloroform protocol [54]. Protein pellets were dried and resuspended in 200 µl of 2X buffer (7 M urea, 2 M thiourea, 4% [w/v] CHAPS and 0.0003% [w/v] bromophenol blue). For 2-D electrophoresis, 100 µl of each sample were diluted to a total volume of 140 µl with 2X buffer, 18.2 mM DTT and 0.5% of IPG buffer solution (pH 3–10) (BIO-RAD) as final concentrations. First dimension was run on IPG strips (pH 3–10 NL, 7 cm; BIO-RAD) in a Protean IEF Cell system (BIO-RAD). As recommended by the manufacturers, a 7 steps program was used: 50 V for 12 h, 250 V for 1 h, 500 V for 1 h, 1000 V for 1 h, 2000 V for 1 h, 8000 V for 1 h [linear ramp] and 8000 V until voltage·time reached 3500 V·h in this step. More than total 12000 V·h were reached in all the cases. Second dimension was run on 12% SDS-PAGE at 0.5 watts/gel for 30 min and then at 1.5 watts/gel until the die-front reached the bottom edge (approximately 2 h) in a Mini-Protean Cell (BIO-RAD). Dual Color Precision Plus Protein Standard (BIO-RAD) was used as molecular weight marker. Gels were stained with Colloidal Blue Staining Kit (Invitrogen). Melanie 2D gel analysis software (version 7.05; Swiss Institute of Bioinformatics, Switzerland) and The EXQuest Spot Cutter (BIO-RAD) were used for imaging the gels, quantify spots and picking the selected spots.

### MALDI Peptide Mass Fingerprinting, Tandem Mass Spectrometry (MS/MS) Analysis and Database Searching

Protein bands were processed automatically in a Proteineer DP (Bruker Daltonics, Bremen, Germany) as described by [55]. The same procedure as described in [56] was then used to process plugs for protein identification.

### Northern and Southern Blotting

The isolation of genomic DNA and total RNA as well as the preparation of DNA probes for Southern and Northern blotting were carried out essentially as described previously by [17].

### Sequence Analyses

The 27 sequences of GMC clade 1 as described by [23] were retrieved from the National Center for Biotechnology Information protein database. As some accession numbers had been superseded, the updated entries were used (see Table 1). In addition, we substituted the *P. pulmonarius* AAO sequence used in the [23] study with the aryl alcohol oxidase (AAO) from *Plerurotus eryngii* [31] because its structure has been described (PDB ID: 3FIM). These two AAO sequences share 95% identity over 593 contiguous amino acids. Additional annotations for sequences were sought by querying the PROSITE database [29] and the SignalP predictor [34].

Retrieval of 500 GMC sequences from the 98 complete Ascomycete sequencing project databases listed on the Hyphal Tip web page (fungalgenomes.org) was carried out by scoring for the presence of both GMC Pfam motifs [28]. The two motifs, pfam05199 (GMC oxred C) and pfam00732 (GMC oxred N) were deemed present at Hidden Markov Model E values of less than 1^−30^ and 1^−50^, respectively, using HMMsearch [57].

### Phylogenetic Analysis

We followed the PHYLIP (version 3.69; [58]) distance method that was used for the analysis presented in Figure 6 of [23], with the addition of GmcA to the 27 clade 1 GMC sequences. In brief, after aligning the sequences using CLUSTAL, columns with more than 50% gaps were removed and 100 data sets were generated for the 548 remaining columns using SEQBOOT and PROTDIST. The most probable tree was generated using FITCH and CONSENSE with 10 randomizations of input order and allowing global rearrangements. The tree graphic was generated using Dentroscope version 2.7.4 [59]. The alignment of GmcA with related sequences was formatted with ESPript [60].

### Partitioning GMC Sequences into Co-factor and Substrate Binding Domains

In order to determine which residues of GmcA comprised each of the two domains, we modeled the sequence onto the PerynAAO structure (PDB ID: 3FIM) using Swiss-Model Workspace [61], [62] as it had the highest similarity to GmcA in the PDB database (98% coverage, 28% identity, E  = 5^−63^). Inspection of the model and comparison of QMEAN scores indicated that the cofactor binding domain was a usable model while the SBD was poorly modeled (with QMEAN scores of 1.8±1.0 and 2.6±1.2, respectively). By mapping the cofactor binding domain of the 3FIM structure onto the GmcA sequence, the amino acids comprising the two domains were identified (cofactor binding domain residues: 1–50, 65–73, 85–96, 111–129, 202–302, 428–444, 512–550, 559–576). This procedure was extended to partition the residues of additional sets of GMCs by editing CLUSTAL alignments with Jalview [63].

## Supporting Information

Figure S1 **Phenotype of the Δ** ***gmcA*** **strain reconstituted with an ectopic copy of** ***gmcA*** **integrated at the** ***pyroA locus*** **.** Wild-type (TN02A3), Δ*gmcA* (BD429) and reconstituted (BD605) strains after 60 hours of culture in MMA supplemented with 80 mM nitrate as the main nitrogen source. The Δ*gmcA* strain shows a marked *fluffy* phenotype in these conditions (see Figure 6 in main text) while the reconstituted strain BD605 displays a conidiating phenotype similar to that of the wild-type. A strain transformed with the empty plasmid was used as a control (BD604; see Materials and Methods). Scale bar  = 2 cm.
(TIF)

Table S1 **Genes identified by mass spectroscopy.**
(DOC)

Table S2 **Compositional characteristics of GMCs and 27 putative Ascomycete GmcA orthologs with BLAST E  = 0.**
(DOC)

Table S3 **Oligonucleotides used in this study.**
(DOC)

Text S1 **Characterization of the GmcA sequence and ortholog search.**
(DOC)
