# Supplementary material for: GmcA Is a Putative Glucose-Methanol-Choline Oxidoreductase Required for the Induction of Asexual Development in Aspergillus nidulans
Source: PLoS One. 2012 Jul 5;7(7):e40292. doi: 10.1371/journal.pone.0040292 (PMC3390393; doi:10.1371/journal.pone.0040292)
Supplement: Table S1 — Genes identified by mass spectroscopy. (DOC) [file pone.0040292.s002.doc]

**Table S1: Genes identified by mass spectroscopy.**

| **Spot** | **Candidate** | **Score/Cover.** | **Locus** | **GO TERM or demonstrated metabolic role** | **Reference** |
| --- | --- | --- | --- | --- | --- |
| **Spots with higher intensity in Δ*flbB* background (V_WT_/V_ΔflbB_ ≤ 0.8)** | | | | | |
| 1 | UTP-glucose-1-phosphate uridylyltransferase | 545/  %64 | *AN9148/*  *galF* | Galactose catabolism. | [1] |
| 2 | Pyruvate decarboxylase | 898/  %64 | *An4888/*  *pdcA* | Pyruvate metabolism. Menadione stress response. | [2–4] |
| 3 | Pyruvate decarboxylase | 871/  %62 | *An4888/*  *pdcA* | Pyruvate metabolism. Menadione stress response. | [2–4] |
| 4 | Glucose-methanol choline oxidoreductase | 448/  %58 | *An8547* | Glucose-methanol-choline oxidoreductase. Menadione stress response. | [4,5] |
| 5 | 6-phosphogluconate dehydrogenase (decarboxylating) | 773/ %70 | *AN3954* | Pentose-phosphate shunt. Response to oxidative stress. | [2] |
| 6 | NADP-specific glutamate dehydrogenase | 829/ %79 | *An4376/ gdhA* | Glutamate and glutamine metabolism. Menadione stress response. | [4,6] |
| **Spots with lower intensity in Δ*flbB* background (V_WT_/V_ΔflbB_ ≥ 1.2)** | | | | | |
| 7 | 2-methylcitrate dehydratase | 379/ %41 | *An6639/*  *mcdB* | Lysine metabolism. | [1] |
| 8 | Enolase (2 phosphoglycerate dehydratase) (A. oryzae) | 1090/  %68 | *An5746/*  *acuN* | Glycolysis and gluconeogenesis. Osmotic and menadione stress response. | [2,4,7] |
| 9 | Citrate synthase | 520/ %41 | *An8275/ citA* | Tricarboxylic acid cycle. | [2,8] |
| 10 | Citrate synthase | 588/  %41 | *An8275/*  *citA* | Tricarboxylic acid cycle. | [2,8] |
| 11 | Citrate synthase | 554/  %49 | *An8275/*  *citA* | Tricarboxylic acid cycle. | [2,8] |
| 12 | Glyceraldehide-3 phosphate dehydrogenase | 614/ %81 | *An8041/ gpdA* | Glycolysis and gluconeogenesis Osmotic stress response | [2,9] |
| 13 | UDPglucose-4 epimerase | 577/  %67 | *An4727/*  *ugeA* | Galactose and galactitol metabolism. Menadione stress response. | [2,4,10] |
| 14 | NADP+-dependent glicerol dehydrogenase | 238/  %48 | *An5563/*  *gldB* | Glycerol metabolism. Osmotolerance. | [2,11] |
| 15 | Fructose-1,6 bisphosphate aldolase | 433/  %73 | *An2875/*  *fbaA* | Glycolysis and gluconeogenesis | [2,4,12] |
| 16 | Not identified | --------- | *----------* | ------------ | --------- |
| 17 | Formate dehydrogenase | 577/ %68 | *An6525/ aciA* | Oxalic acid metabolism. Menadione stress response. | [2,4,13] |
| 18 | Aspartate transaminase | 464/ %54 | *An6048* | Amino acid metabolism. | [14] |
| 19 | Transaldolase | 718/ %64 | *An0240/*  *pppA* | Pentose-phosphate shunt. Oxidative stress response. | [1,2,4] |
| 20 | Triose-phosphate isomerase | 932/ %91 | *An6900/*  *tpiA* | Glycolysis and gluconeogenesis. | [2,15] |
| 21 | Ribose-5-phosphate isomerase (ribose/galactose isomerase) | 304/ %66 | *An5907* | Pentose phosphate pathway. Menadione stress response. | [1,1,4] |

Reference List

1. Flipphi M, Sun J, Robellet X, Karaffa L, Fekete E, Zeng AP, Kubiecek CP (2009) Biodiversity and evolution of primary carbon metabolism in *Aspergillus nidulans* and other Aspergillus spp. Fungal Genet Biol 46 Suppl 1: S19-S44.

2. David H, Ozcelik IS, Hofmann G, Nielsen J (2008) Analysis of *Aspergillus nidulans* metabolism at the genome-scale. BMC Genomics 9: 163. 1471-2164-9-163 [pii];10.1186/1471-2164-9-163 [doi].

3. Lockington RA, Borlace GN, Kelly JM (1997) Pyruvate decarboxylase and anaerobic survival in *Aspergillus nidulans*. Gene 191: 61-67. S0378111997000322 [pii].

4. Pusztahelyi T, Klement E, Szajli E, Klem J, Miskei M, Karanyi Z, Emri T, Kovacs S, Orosz G, Kovacs KL, Medzihradszky KF, Prade RA, Pocsi I (2011) Comparison of transcriptional and translational changes caused by long-term menadione exposure in *Aspergillus nidulans*. Fungal Genet Biol 48: 92-103. S1087-1845(10)00169-6 [pii];10.1016/j.fgb.2010.08.006 [doi].

5. Levasseur A, Piumi F, Coutinho PM, Rancurel C, Asther M, Delattre M, Henrissat B, Pontarotti P, Asther M, Record E (2008) FOLy: an integrated database for the classification and functional annotation of fungal oxidoreductases potentially involved in the degradation of lignin and related aromatic compounds. Fungal Genet Biol 45: 638-645. S1087-1845(08)00006-6 [pii];10.1016/j.fgb.2008.01.004 [doi].

6. Hawkins AR, Gurr SJ, Montague P, Kinghorn JR (1989) Nucleotide sequence and regulation of expression of the *Aspergillus nidulans gdhA* gene encoding NADP dependent glutamate dehydrogenase. Mol Gen Genet 218: 105-111.

7. Hynes MJ, Szewczyk E, Murray SL, Suzuki Y, Davis MA, Sealy-Lewis HM (2007) Transcriptional control of gluconeogenesis in *Aspergillus nidulans*. Genetics 176: 139-150. genetics.107.070904 [pii];10.1534/genetics.107.070904 [doi].

8. Murray SL, Hynes MJ (2010) Metabolic and developmental effects resulting from deletion of the *citA* gene encoding citrate synthase in *Aspergillus nidulans*. Eukaryot Cell 9: 656-666. EC.00373-09 [pii];10.1128/EC.00373-09 [doi].

9. Punt PJ, Dingemanse MA, Jacobs-Meijsing BJ, Pouwels PH, van den Hondel CA (1988) Isolation and characterization of the glyceraldehyde-3-phosphate dehydrogenase gene of *Aspergillus nidulans*. Gene 69: 49-57. 0378-1119(88)90377-0 [pii].

10. El-Ganiny AM, Sheoran I, Sanders DA, Kaminskyj SG (2010) *Aspergillus nidulans* UDP-glucose-4-epimerase UgeA has multiple roles in wall architecture, hyphal morphogenesis, and asexual development. Fungal Genet Biol 47: 629-635. S1087-1845(10)00043-5 [pii];10.1016/j.fgb.2010.03.002 [doi].

11. de Vries RP, Flitter SJ, van de Vondervoort PJ, Chaveroche MK, Fontaine T, Fillinger S, Ruijter GJ, d'Enfert C, Visser J (2003) Glycerol dehydrogenase, encoded by *gldB* is essential for osmotolerance in *Aspergillus nidulans*. Mol Microbiol 49: 131-141. 3554 [pii].

12. Roumelioti K, Vangelatos I, Sophianopoulou V (2010) A cryptic role of a glycolytic-gluconeogenic enzyme (aldolase) in amino acid transporter turnover in *Aspergillus nidulans*. Fungal Genet Biol 47: 254-267. S1087-1845(09)00215-1 [pii];10.1016/j.fgb.2009.12.004 [doi].

13. Saleeba JA, Cobbett CS, Hynes MJ (1992) Characterization of the *amdA*-regulated *aciA* gene of *Aspergillus nidulans*. Mol Gen Genet 235: 349-358.

14. Kim Y, Islam N, Moss BJ, Nandakumar MP, Marten MR (2011) Autophagy induced by rapamycin and carbon-starvation have distinct proteome profiles in *Aspergillus nidulans*. Biotechnol Bioeng . 10.1002/bit.23223 [doi].

15. McKnight GL, O'Hara PJ, Parker ML (1986) Nucleotide sequence of the triosephosphate isomerase gene from *Aspergillus nidulans*: implications for a differential loss of introns. Cell 46: 143-147. 0092-8674(86)90868-8 [pii].
