# Supplementary material for: GmcA Is a Putative Glucose-Methanol-Choline Oxidoreductase Required for the Induction of Asexual Development in Aspergillus nidulans
Source: PLoS One. 2012 Jul 5;7(7):e40292. doi: 10.1371/journal.pone.0040292 (PMC3390393; doi:10.1371/journal.pone.0040292)
Supplement: Table S2 — Compositional characteristics of GMCs and 27 putative Ascomycete GmcA orthologs with BLAST E = 0. (DOC) [file pone.0040292.s003.doc]

**Table S2: Compositional characteristics of GMCs and 27 putative Ascomycete GmcA orthologs with BLAST E = 0.**

**
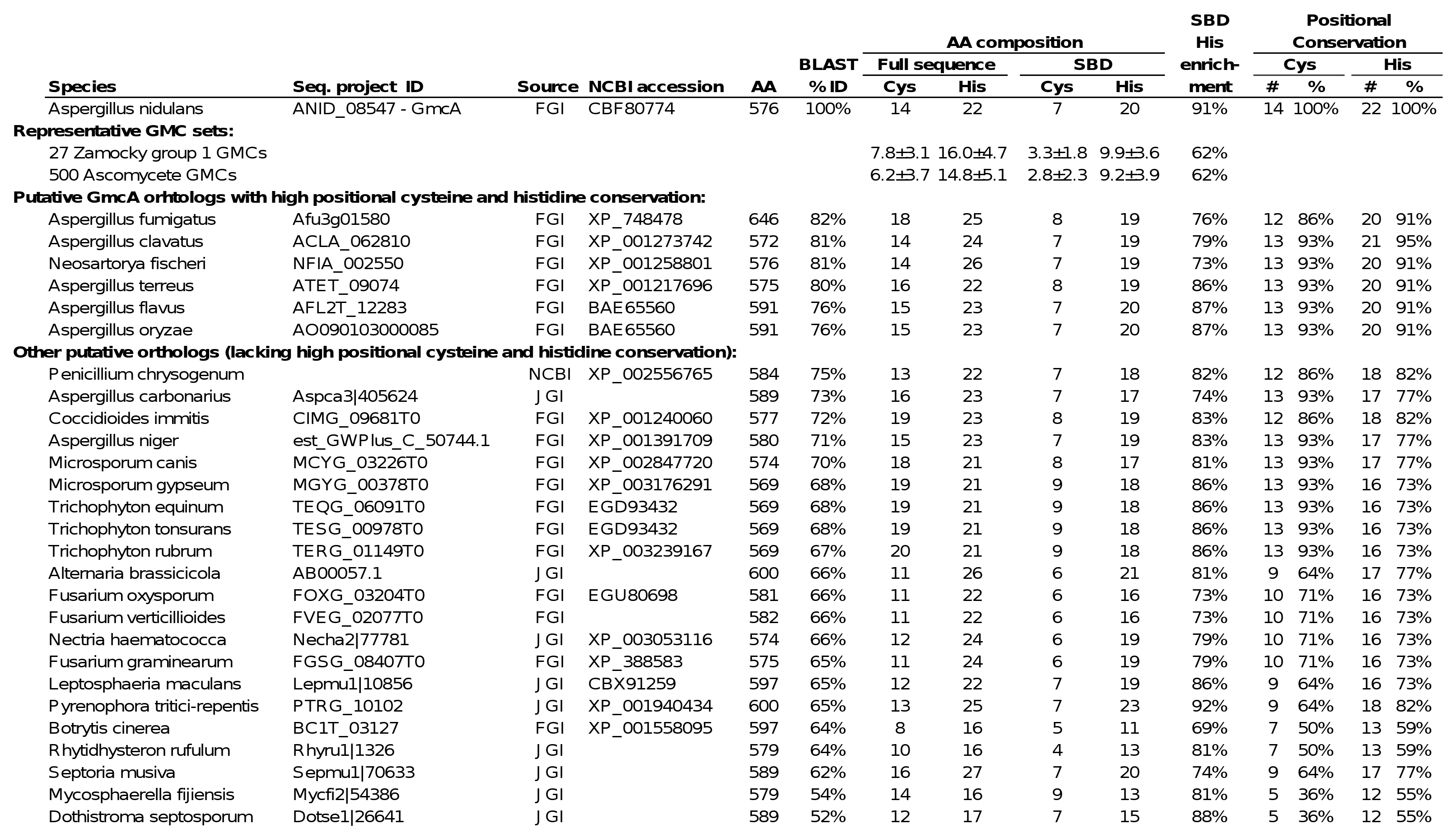
**
