# Supplementary material for: GmcA Is a Putative Glucose-Methanol-Choline Oxidoreductase Required for the Induction of Asexual Development in Aspergillus nidulans
Source: PLoS One. 2012 Jul 5;7(7):e40292. doi: 10.1371/journal.pone.0040292 (PMC3390393; doi:10.1371/journal.pone.0040292)
Supplement: Table S3 — Oligonucleotides used in this study. (DOC) [file pone.0040292.s004.doc]

**Table S3: Oligonucleotides used in this study.**

| **Name** | **Sequence 5´-3´** | **Objective** |
| --- | --- | --- |
| gmcA-PP1 | CCTCGTGCATTCAACAACCTAAGGGGG | Null Δ*gmcA* mutant |
| gmcA-PP2 | TTTTGCAGTGATTTCAGTGCGAGGACGG | Null Δ*gmcA* mutant |
| gmcA-SMP1 | CCGTCCTCGCACTGAAATCACTGCAAAA accggtcgcctcaaacaatgctct | Null Δ*gmcA* mutant |
| gmcA-GFP2 | CCACTTTTGCGACTCCAGGTCTCGGCCTA GTCTGAGAGGAGGCACTGATGCG | Null Δ*gmcA*, *gmcA::gfp* and *gmcA::3ha* strains |
| gmcA-GSP3 | TAGGCCGAGACCTGGAGTCGCAAAAGTGG | Null Δ*gmcA*, *gmcA::gfp* and *gmcA::3ha* strains |
| gmcA-GSP4 | GCTTCACCATCACGACAGTAACACACCG | Null Δ*gmcA*, *gmcA::gfp* and *gmcA::3ha* strains |
| gmcA-GFP1 | GCGCGGACTTTATCAAGGAGGAGTGG GGAGCTGGTGCAGGCGCTGGAGCC | *gmcA::gfp* and *gmcA::3ha* strains |
| gmcA-GSP1 | CTTTTGGCTTTCGTCATCCCCGC | *gmcA::gfp and gmcA::3ha* strains |
| gmcA-GSP2 | CCACTCCTCCTTGATAAAGTCCGCGC | *gmcA::gfp and gmcA::3ha* strains |
| alcA-gmcA-Up | CGGGATCCCCTCGCACTGAAATCACTGC | *gmcA::gfp* overexpression strains |
| flbF-gfpFP-alcA | GGAATTCCTCATTTGTATAGTTCATCCA TGCCATGTGT | *gmcA::gfp* overexpression strains |
| gmcAseq | CCCCATCGCTGACCG | Sequencing of *gmcA* cDNA |
| SP6 | GATTTAGGTGACACTATA | Sequencing of *gmcA* cDNA |
| T7 | TAATACGACTCACTATAGGGAGA | Sequencing of *gmcA* cDNA |
