# Supplementary material for: GmcA Is a Putative Glucose-Methanol-Choline Oxidoreductase Required for the Induction of Asexual Development in Aspergillus nidulans
Source: PLoS One. 2012 Jul 5;7(7):e40292. doi: 10.1371/journal.pone.0040292 (PMC3390393; doi:10.1371/journal.pone.0040292)
Supplement: Text S1 — Characterization of the GmcA sequence and ortholog search. (DOC) [file pone.0040292.s005.doc]

**Text S1: Characterization of the GmcA sequence and ortholog search.**

Composition analysis of GmcA and orthologs revealed two amino acid enrichments that may play a role in the specific function of GmcA. First, GmcA is enriched in cysteines, with 14 being distributed equally between the two structural domains. Second, the SBD of GmcA is substantially enriched in histidines, with 20 of 22 histidines (91%) concentrated there. To establish a set of putative GmcA orthologs, sequences with BLAST E values of zero were derived from the set of 500 putative ascomycota GMCs. Of 26 sequences, none was predicted to have a signal sequence, in agreement with GmcA. To further rank these orthologs, we evaluated the positional conservation of cysteines and histidine residues in relation to GmcA. A higher confidence set of six orthologs exhibited positional conservation equal to or greater than 85% for both of these residues (Table S2). Conversely, the most similar GMC outside of the ascomycota, a putative choline dehydrogenase from the bacteria *Runella slithyformis* (NCBI accession YP_004654190), is only 36% identical. Moreover, there was no correlation of the cysteines and histidine content and distribution between GmcA and this sequence. Thus the GMC type defined by GmcA is restricted to fungi, specifically the ascomycota.
